# Supplementary material for: Early Age- and Sex-Dependent Regulation of Astrocyte-Mediated Glutamatergic Synapse Elimination in the Rat Prefrontal Cortex: Establishing an Organotypic Brain Slice Culture Investigating Tool
Source: Cells. 2023 Dec 4;12(23):2761. doi: 10.3390/cells12232761 (PMC10705965; doi:10.3390/cells12232761)
Supplement: Supplementary file 1 [file cells-12-02761-s001.zip › cells-2710937-supplementary.pdf]

Supplementary Figure S1 – Uncropped Western Blots.

Male pups – MEGF10, β-actin, PSD95, Synaptophysin, Cofilin

Fig.1B

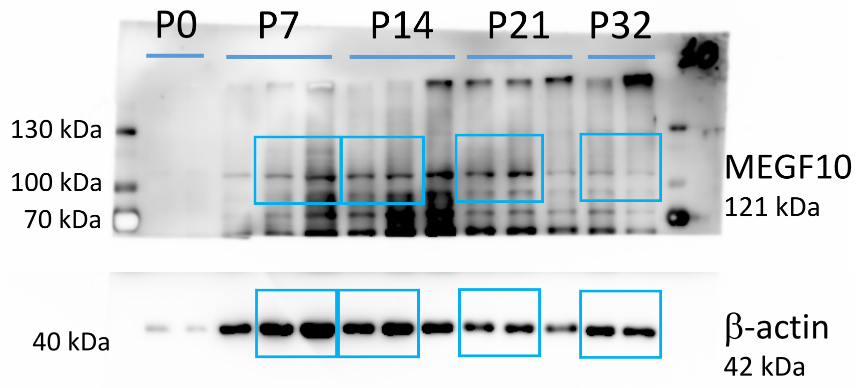

Fig.1D

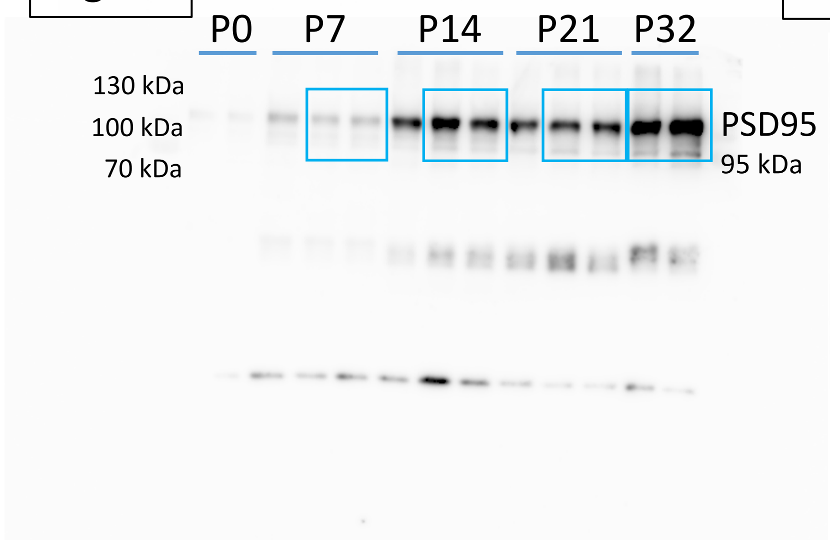

Fig.1F

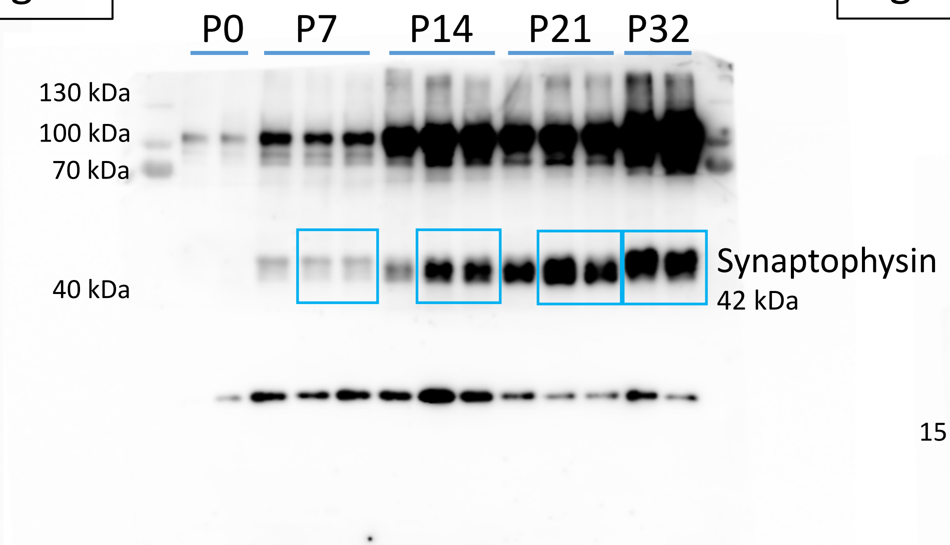

Fig.1D &F

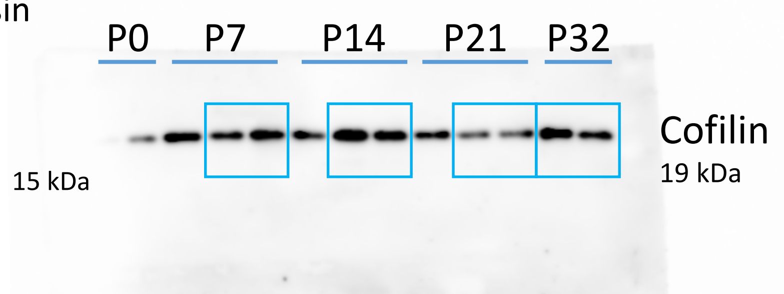

To detect each protein, proper exposure time were applied. Protein analysis was carried out on original images.

Female pups – MEGF10, β-actin, PSD95, Synaptophysin, Cofilin

Fig.1H

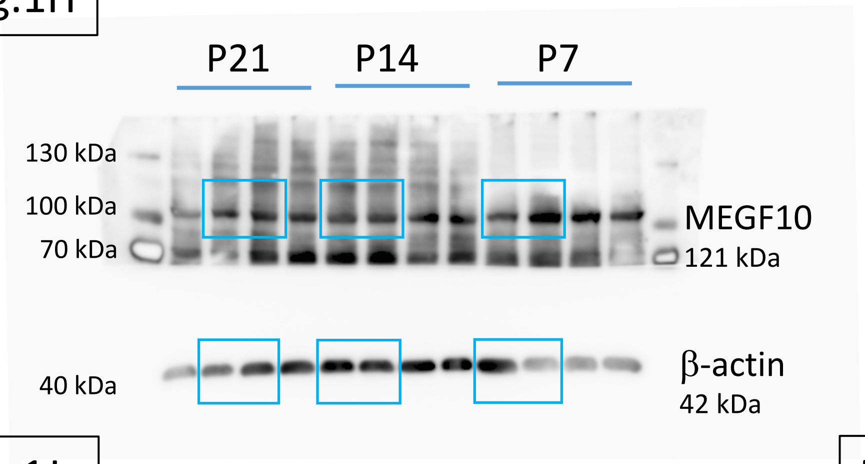

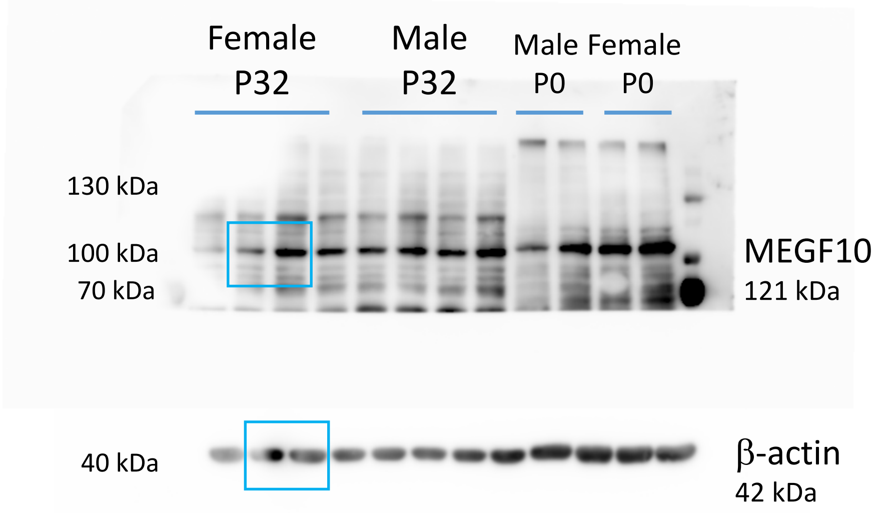

Fig.1J

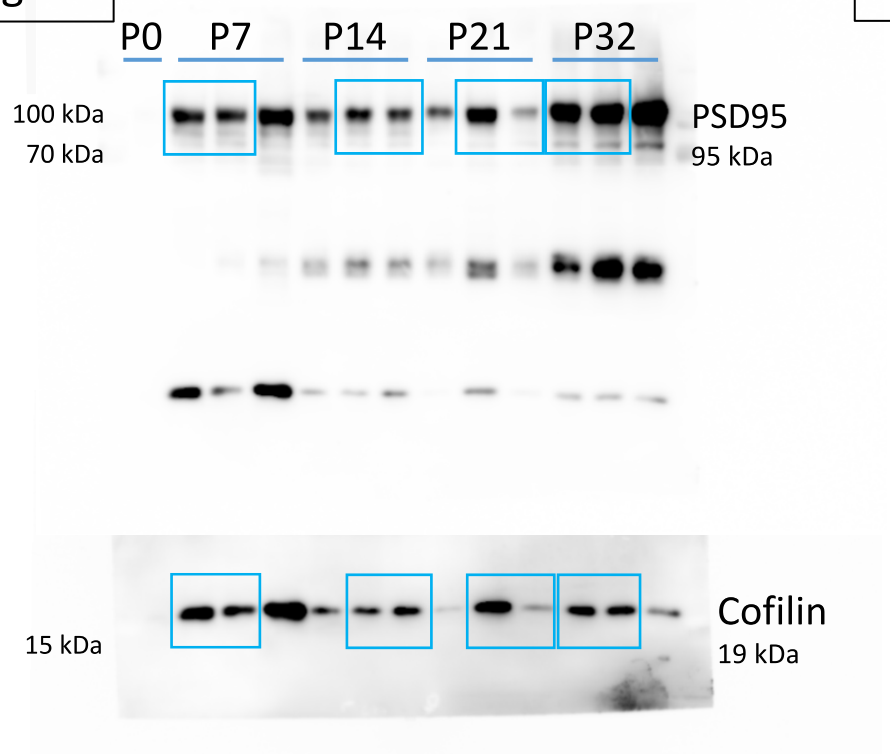

Fig.1L

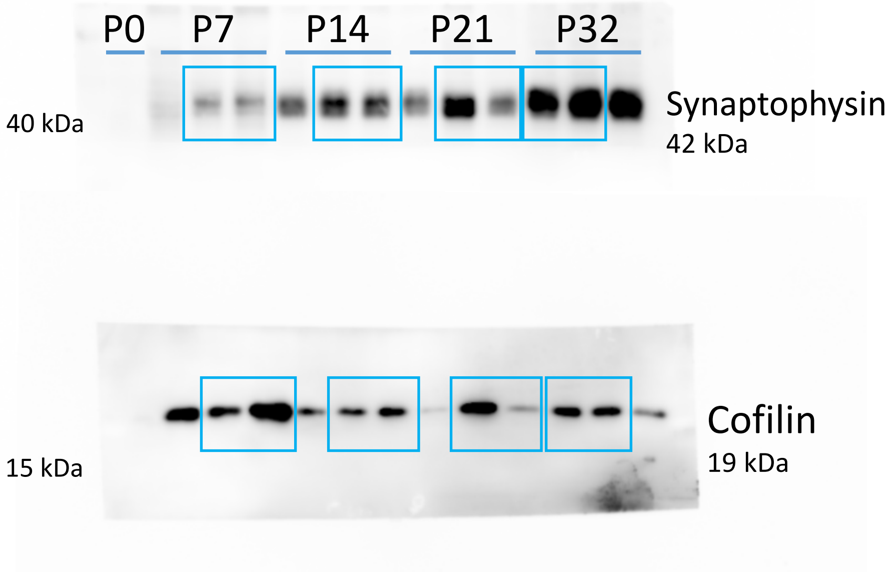

To detect each protein, proper exposure time were applied. Protein analysis was carried out on original images.

Supplementary Figure 4 – Uncropped Western Blots

male OBSCs– MEGF10 & β-actin

Fig.4A

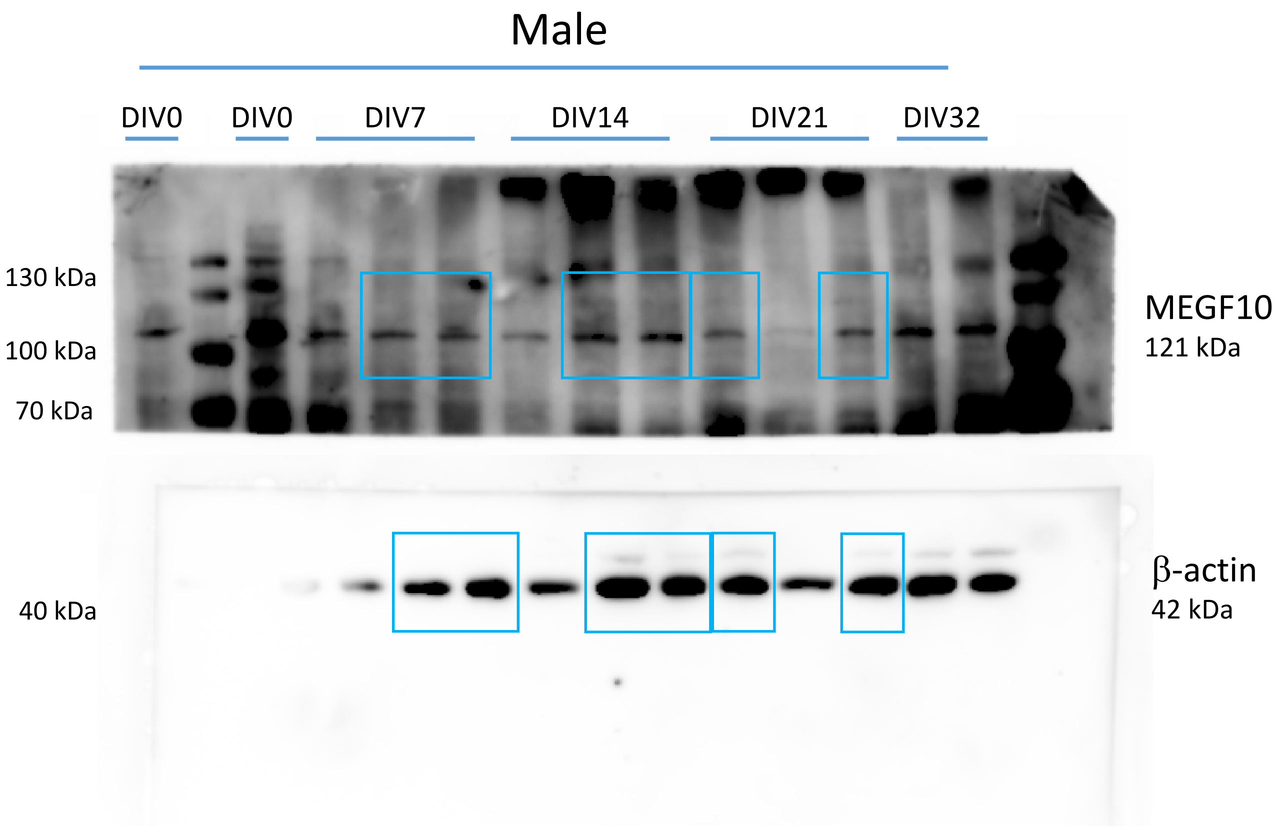

To detect each protein, proper exposure time were applied. Protein analysis was carried out on original images.

female OBSCs– MEGF10 & β-actin

Fig.4E

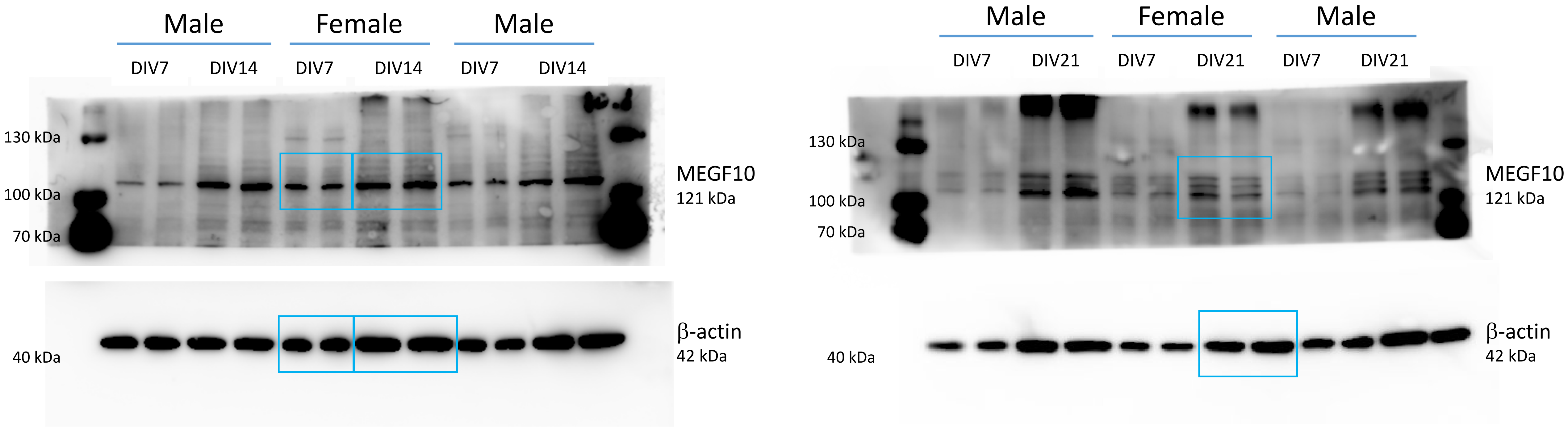

To detect each protein, proper exposure time were applied. Protein analysis was carried out on original images.

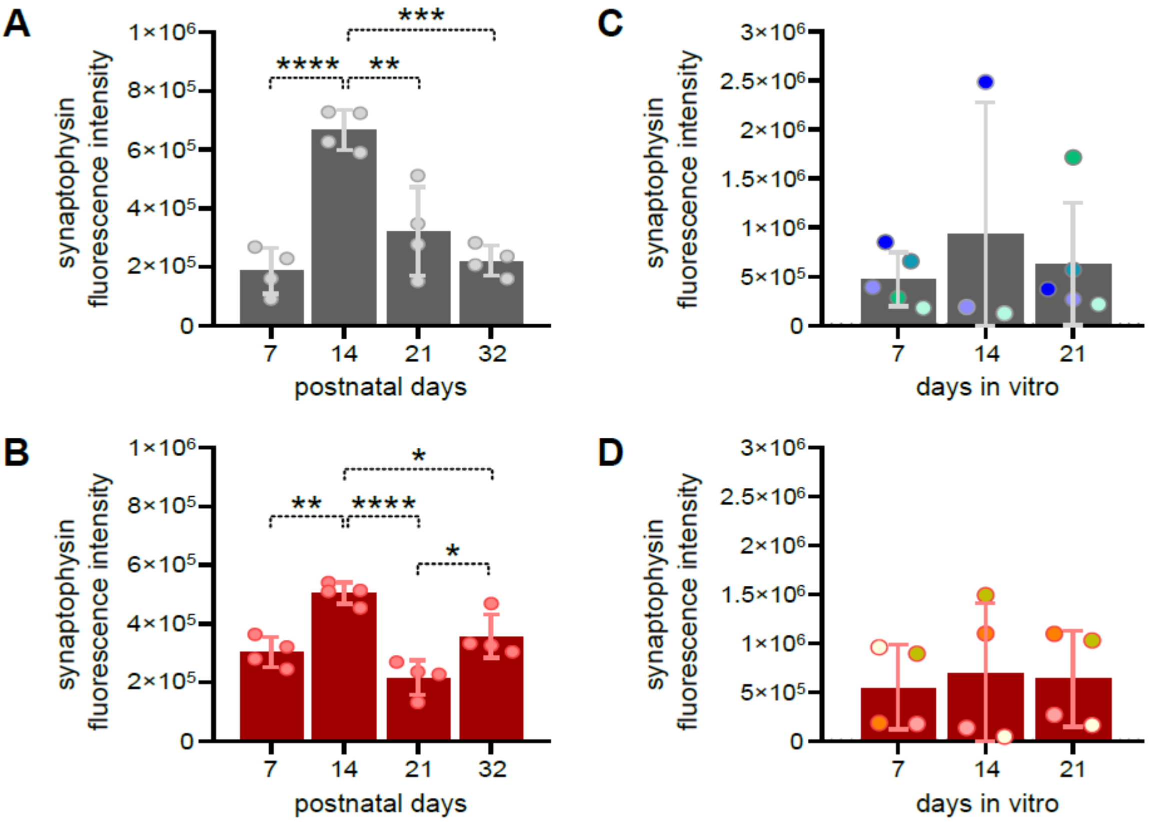

Supplementary Figure S2—Quantification of age- and sex-dependent changes in synaptophysin fluorescent intensiy in vivo. (A,B) and in OBSCs (C,D) in males (A,C) and females (B,D). (A) Quantification of synap-tophysin fluorescent intensity in the PFC at different postnatal developmental stages in male littermates. Each dot represents the average data of 6 pictures from at least 2 slices for each animal: n= 4 animals. One-way ANOVA, Tukey´s multiple comparison, ** $p < 0.01$; *** $p < 0.001$; **** $p < 0.0001$. (B) Quanti-fication of synaptophysin fluorescent intensity in the PFC at different postnatal developmental stages of female littermates. Each dot represents the average data of 6 pictures from at least 2 slices for each animal: n= 4 animals. One-way ANOVA, Tukey´s multiple comparison, * $p < 0.05$; ** $p < 0.01$; **** $p < 0.0001$. (C) Quantification of synaptophysin fluorescent intensity in male-derived OBSCs at different timepoints. Each dot represents one animal, n = 3–5 animals. Sta-tistical analysis was performed via one-way ANOVA repeated measures, with Tukey´s multiple comparison. (D) Quantification of synaptophysin fluorescent intensity in female-derived OBSCs at different timepoints. Each dot represents one animal, n = 4 animals. Statistical analysis was per-formed via one-way ANOVA repeated measures, with Tukey´s multiple comparison.
